# Morphological Analysis of the Mandibular Lingula and Its Relation to Antilingula Using Cone-Beam Computed Tomography in the Saudi Population

**DOI:** 10.3390/diagnostics13030419

**Published:** 2023-01-23

**Authors:** Guna Shekhar Madiraju, Rohini Mohan

**Affiliations:** 1 Department of Preventive Dental Sciences, College of Dentistry, King Faisal University, Al Ahsa 31982, Saudi Arabia; 2 Port Talbot Research Centre, Community Dental Services, Swansea Bay University Health Board, Port Talbot SA12 7BJ, UK

**Keywords:** mandible, lingula, antilingula, morphology, cone-beam computed tomography

## Abstract

Adequate knowledge of the anatomy of the medial aspect of the mandibular ramus is essential in order to prevent inferior alveolar nerve anesthetic failure and nerve injuries associated with mandibular ramal surgical procedures. The purpose of this study was to assess the morphology of the lingula and analyze the correlation of presence probability between the lingula and antilingula using cone-beam computed tomography (CBCT). Three-dimensional images reconstructed from mandibular CBCT images of 125 Saudi individuals (250 sides), aged 16–36 years, were retrospectively evaluated for distribution in the shape of the lingulae. Additionally, the presence probability of the lingula and antilingula was assessed with respect to gender and the mandible (unilateral and bilateral), and correlation between the variables was analyzed. A nodular shape of the lingula was most commonly found (37.6%) followed by a triangular shape (30%). No significant differences were observed between the genders with regard to the presence of the lingula (*p* = 0.108) and antilingula (*p* = 0.530). Gender was non-significantly correlated with the presence of the lingula and antilingula, whilst the presence of lingula showed a significant but weak correlation with that of the antilingula (r_s_ = 0.221; *p* = 0.000). The present study provides information regarding morphological variations of the mandibular lingula in the Saudi population. The presence of the lingula was unassociated with that of the antilingula and hence consideration of the antilingula as the absolute landmark for mandibular ramus surgical procedures seems insufficient.

## 1. Introduction

The mandibular lingula is a tongue-shaped bony projection located on the medial aspect of the ramus in close proximity to the mandibular foramen, while the antilingula is a bony prominence below the sigmoid notch on the lateral side of the mandibular ramus [1]. The mandibular lingula is an important landmark for many maxillofacial surgical procedures involving the mandibular ramus and forms the basis of horizontal cut in sagittal split ramus osteotomy (SSRO) because of its relationship to the inferior alveolar nerve (IAN). The advantage of SSRO is that the surgical access to the mandible is intraoral, which lowers the risk of injury to the facial nerve. The antilingula has been used to approximately analyze the anatomical position of the lingula during surgical procedures, especially IVRO, to prevent damage to the inferior alveolar neurovascular bundle [2,3]. The position of the lingula constantly changes with age during the growth phase of the mandible, and a high degree of variation in the morphometrics of the lingula has been reported [4]. Hence, adequate knowledge of the anatomy of the medial aspect of the mandibular ramus including the shape of the lingula is imperative in order to prevent inferior alveolar anesthetic failure, nerve injuries and complications associated with mandibular ramal surgical procedures [5].

Previous studies investigating the morphometrics of the lingula have used dry mandible specimens and panoramic radiographs. However, cone-beam computed tomography (CBCT) has been reported to be superior to panoramic radiography in visualizing the mandibular landmarks owing to its highly accurate images and precise location of anatomic structures. Whilst many authors have used the lingula as an ideal anatomical landmark to determine the position of mandibular foramen, variables such as gender, race or ethnicity and the shape of the lingula were not considered. Studies have reported different morphological types of lingula among different populations and races [5,6], and the reason for variations in shape is unknown. The data on morphometric analysis of the lingula in the Saudi population are scarce in the literature. Hence, the present study aimed to assess the prevalence of different shapes of lingula and evaluate the height of the lingula in a sample of the Saudi population from the eastern province of Saudi Arabia. Additionally, the presence probability of lingula and antilingula was analyzed on mandibular images obtained using CBCT.

## 2. Materials and Methods

This retrospective observational study comprised CBCT images of 855 Saudi patients who presented to the university’s dental unit between Sep 2019 and Nov 2021. CBCT imaging was advised in these patients for various reasons including orthodontics, oral pathology, maxillofacial surgery, endodontics and dental implants. The inclusion criteria were subjects aged between 16–36 years and good quality of CBCT mandibular images. The exclusion criteria involved the presence of bone pathology, a history of systemic disease affecting osseous metabolism, a history of posterior mandibular fracture, previous orthodontic treatment periapical pathology, and supernumerary teeth. Finally, 125 (68 males and 57 females) out of 855 CBCT images were included in the study. The study protocol was reviewed and approved by the Institutional Review Board and the study was conducted in accordance with the ethical standards laid down in 1964 Declaration of Helsinki.

All of the CBCT scans were acquired with an i-CAT Vision QTM CBCT unit (Imaging Sciences International, Inc., Hatfield, PA, USA) operating at 120 kVp, 5 mA, with an exposure time of 2–7 s and a field of view of 13 cm with a 0.25 mm voxel size. Once the three-dimensional (3D) images of every sample had been processed, the data were analyzed with i-CAT Vision software (Imaging Sciences International). The DICOM images were converted into 3D surface models of the mandibles and the study variables were analyzed on the reconstructed 3D images of each mandible of the entire sample using a 17-inch, high-resolution colour screen monitor. The image contrast and brightness were adjusted (using the image processing tool in the software) to ensure optimal visualization of the anatomical landmarks.

**Figure 1:**
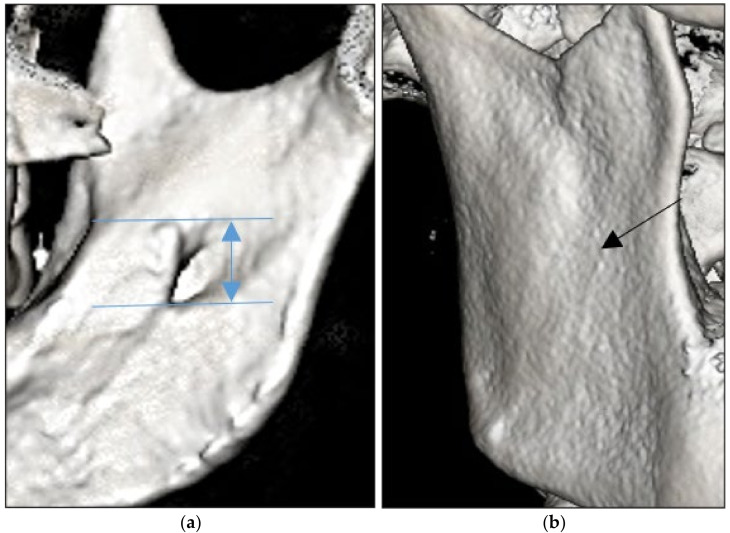
(**a**) Height of the lingula and (**b**) presence of the antilingula (arrow).

The lingula point was recorded as the most superior point of the lingula (tip), independently of its shape. The height of the lingula was measured as the vertical distance from the tip of the lingula to the lowest point of the mandibular foramen (MF), on both the right and left sides of the mandible (Figure 1a). The antilingula was identified as the most prominent point on the lateral surface of the mandibular ramus (Figure 1b). The shapes of the lingulae were classified as triangular, truncated, nodular and assimilated (which indicated the absence of the lingula) based on the classification proposed by Tuli et al. (2000) [6]. The reconstructed 3D images were examined for the presence or absence of antilingula. Analyses of all of the CBCT images were carried out by the same investigator, who was pre-calibrated before the study. To evaluate the intra-observer agreement using the intraclass correlation coefficient (ICC), the observations were repeated three times in 10% of the sample, with an interval of two weeks. The ICC showed excellent agreement between the measurements (≥0.95).

The statistical analysis was completed using the Statistical Package for the Social Sciences (IBM SPSS v.20 software for Windows [IBM Corp, Armonk, NY, USA]). Descriptive analysis of the data was carried out and a *p* value of 0.05 was considered statistically significant. The mean and respective standard deviations (SDs) were calculated for the height of the lingula. Due to a lack of normal distribution, the Mann–Whitney U non-parametric test for independent samples and the Wilcoxon test for related samples were applied. The chi-squared test and Spearman’s correlation test were used to determine the relationship between gender and the presence of the lingula and antilingula.

## 3. Results

**Figure 2:**
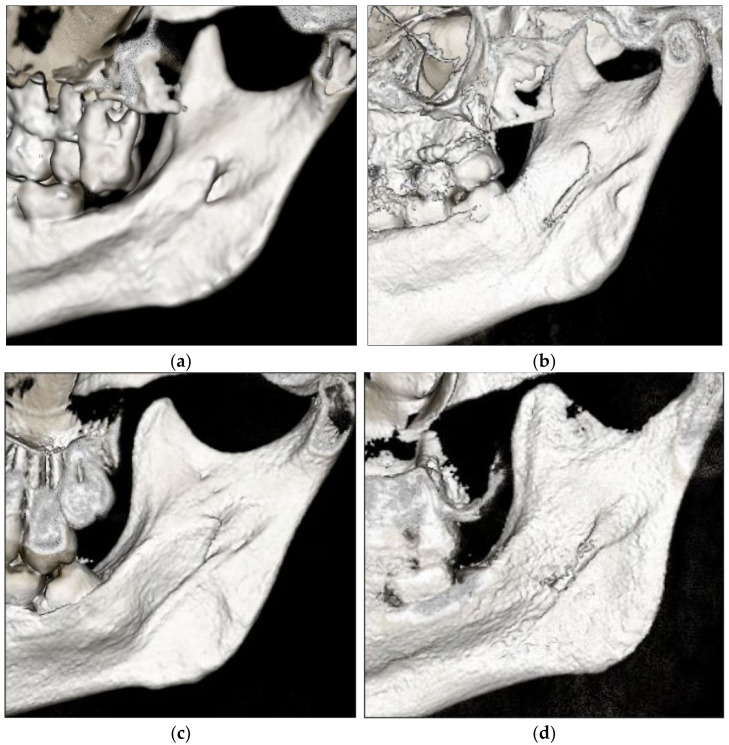
Different shapes of the lingula: (**a**) nodular, (**b**) truncated, (**c**) triangular and (**d**) assimilated.

CBCT scans of 125 patients (*n* = 250 sides) from the radiology archives were eligible to be included in the study. The study subjects consisted of 68 males (54.4%) and 57 females (45.6%), and the mean (SD) age of the study sample was 24.2 ± 4.71 years. The most common shape of the lingula observed was nodular (37.6%), followed by triangular (30%), truncated (22%) and assimilated types (10.4%) (Figure 2). A bilateral shape (43.2%) was found less often than a unilateral one (56.8%). The lingulae were found bilaterally as nodular in 40 sides (37%), triangular in 36 sides (33.3%), truncated in 22 sides (20.4%) and assimilated in 10 sides (9.3%) (Table 1). When the distribution of the lingula shape was compared between the females and males (Table 2), a statistical difference between the genders was noted only in the nodular type (*p* = 0.016).

The overall mean height of the lingula (in mm) in both of the genders was 7.73 ± 0.44, and it was 7.83 ± 0.47 on the right side and 7.63 ± 0.41 on the left side. Statistically significant differences between the genders are shown in Table 3. Within the gender variable, significant differences in the height of the lingula between the right and left sides were observed in both males (*p* = 0.000; z = −9.471) and females (*p* = 0.000; z = −4.240).

Of the 250 sides (CBCTs), the lingula was noted as absent in 10.4% (*n* = 26) of the sample. The lingula was observed in males on 118 sides (86.7%) and on 106 sides (93%) in females. The antilingula was indicated as absent on 176 sides (70.4%), while it was seen on 38 sides (27.9%) and 36 sides (31.6%) in males and females, respectively. There were no statistically significant differences between the genders with regard to the presence of the lingula (*p* = 0.108) and antilingula (*p* = 0.530) (Table 4).

The distribution of the lingula and antilingula by the variable of gender and mandibular side (bilateral vs. unilateral) is shown in the Table 5. The bilateral presence of the lingula was observed on 98 sides (90.7%), and the bilateral absence of the lingula was observed on 10 sides (9.3%). The bilateral presence of the antilingula was observed on 36 sides (20.7%), and the bilateral absence of the antilingula was observed on 138 sides (79.3%). The presence of the lingula and antilingula showed no significant differences in the variable of gender (*p* > 0.05). Regarding the Spearman’s correlation (r_s_) analysis of gender, the presence of the lingula showed a weak correlation with that of the antilingula (r_s_ = 0.221; *p* = 0.000). Gender was non-significantly correlated with the presence of the lingula (r_s_ = −0.101; *p* = 0.110) and antilingula (r_s_ = −0.040; *p* = 0.532).

## 4. Discussion

The lingula has been considered as an important clinical landmark during surgical procedures involving IAN block and the mandibular ramus. Most data available on the anatomical shape, position and presence probability of the lingula or antilingula have mostly been based on the measurements of dry human mandibles. However, in most cases, dry mandibles cannot provide adequate information on sex, age or race [7]. CBCT offers high-resolution 3D images for proper visualization of mandibular morphology and measurement accuracy, which corresponds closely to the actual size of the object. In the present study, 3D images were reconstructed with a slice thickness of 0.2 mm and a voxel size of 0.2 mm, which can be considered acceptable. To the best of our knowledge, this is the first study to investigate the distribution of shapes of the lingula and analyze the presence of the lingula and antilingula in a sample of the Saudi population.

Variations in the morphological types of the lingula among different populations and races have been reported [5,6,8,9,10,11,12,13,14,15,16] (Table 6). The triangular variant was more prevalent in the Indian population [6,13] and truncated variant was common in Thai [5], South African [11], Brazilian [10] and Italian [16] population groups. The present study revealed that the nodular shape of the lingula was most frequently found (37.6%) which corroborates with the findings of other studies in Turkish [8,9] and Korean populations [12]. The least prevalent lingula noted was the assimilated variant (10%) which is in line with most studies in the literature [8,12,14,15,16]. Whilst most studies have revealed that lingulae with bilaterally similar shapes are common, the possible coexistence of different morphologies of the lingula on the two mandibular sides in the same subject has also been highlighted [17]. The present study observed that the shape of the lingulae were symmetrical on both sides in 43.2% of the mandibles, which is lower than that reported by previous studies [8,9,14]. All types of lingulae were observed to be more prevalent unilaterally. With respect to gender, the current study revealed that males showed a predominance of triangular lingulae (*p* = 0.244), whilst the nodular variant was significantly more prevalent in females (*p* = 0.016), a finding in accordance with that of Ahn et al. (2020) in the Korean population [14].

The lingula and antilingula have been considered as crucial landmarks in mandibular surgery. This study examined the presence probability of the lingula and antilingula with respect to gender and the mandible (unilateral and bilateral). The assimilated shape type has been indicated as the absence of the lingula [3,6]. Previous studies on dry mandibles [15,16] and CBCT [3,8,12,14] have reported considerable differences in the presence probability of the lingula. A study in the Turkish population [9] reported that the lingula was absent in 26.2% of the patients, which was comparatively higher than in other studies. In the present study, absence of the lingula was observed in 10.4% of the mandibular sides, which concurs with the findings of Ahn et al. (2020) [14]. These differences might be attributed to the age, ethnicity and skeletal pattern of the patients. Moreover, the use of CBCT in our study provided real-time image visualization of mandibular morphology compared to variable preservation of dry mandible specimens.

The antilingula has been considered as a highly variable anatomical landmark and different opinions have been suggested regarding the presence probability of the antilingula and the reliability of its use as the reference point for mandibular surgery [18,19,20]. The bone prominence of the antilingula has been related to the musculotendinous apparatus attached to that portion of the mandible rather than to the entrance of the inferior alveolar nerve [18]. Hsiao et al. (2020) [3] had reported that the antilingula was present in 81% of the subjects and 67.8% of those present were bilateral. Zhao et al. (2019) [2] in a CBCT study reported the incidence of the antilingula in 57% of patients, more common on the right side compared to the left and it was noted bilaterally in 32% of patients. In the present study, no significant differences were noted in the presence probability of the lingula and antilingula with respect to gender and the mandible (unilateral and bilateral) (*p* > 0.05). Furthermore, the presence of the antilingula was observed in 29.6% of the sides, which was considerably lower than that of the lingula (89.6%). The present study objectively investigated the CBCT scans of the mandible for the presence of the antilingula and correlated it with the lingula and gender. The Spearman’s correlation analysis revealed that gender was non-significantly correlated with the presence of the lingula and antilingula. The presence of the antilingula showed a weak correlation with that of the lingula. Hence, the use of the antilingula as an absolute landmark for approximating the position of the lingula in mandibular ramus surgeries could be insufficient. Since variations in the size of the mandibular ramus may exist between patients, vertical and horizontal relationships between the lingula and antilingula should be explored further.

Variations in the height of the lingula exist in different population groups [8]. The height of the lingula, in the present study, was measured as the vertical distance from the tip of the lingula to the lowest point of the MF. The overall mean height of the lingula (7.73 ± 0.44 mm), in the present study, was comparatively lower than that reported in other population groups [9,15]. Males and right mandible hemiarches showed a greater mean height of the lingulae which was consistent with those observed in the literature [8,9], whilst Ozalp et al. (2020) [15] in a study on dry mandibles reported higher mean values on the left side, with no significant differences between the genders.

One shortcoming of our study was that the skeletal patterns of the study individuals were not analyzed. Comparison of the present data with similar population groups is limited due to the paucity of information in Arab the population. The use of a population sample derived from those visiting a dental health clinical complex in a university setting may not represent the general population and hence limits the generalizability of the findings in the present study.

## 5. Conclusions

This is the first study investigating the anatomical shape of the lingula and the presence probability of the lingula and antilingula on mandibular images obtained using CBCT in a sample of the Saudi population from the eastern province of Saudi Arabia. The nodular shape of the lingula was the most common type observed in this study. The presence of the antilingula was observed in only 29.6% of the mandibular sides and showed a weak correlation with the lingula. No significant differences were noted in the presence probability of the lingula and antilingula with respect to gender and the mandibular side (unilateral and bilateral). The findings of this research contribute to the current knowledge and research on the morphometric features of the mandibular lingula in the study population. Further studies involving larger population groups are needed to investigate the effect of variables such as craniometry and ethnicity on the mandibular lingula morphometrics.

## Figures and Tables

**Table 1:** Distribution and incidence (in parentheses) of the lingula in males and females.

	Male		Female		Bilateral (*n* = 216)	Unilateral (*n* = 284)		Total
	Bilateral	Unilateral	Bilateral	Unilateral		Right	Left	
Nodular	09	24	11	30	40 (37%)	24	30	94 (37.6%)
Triangular	11	23	07	16	36 (33.3%)	17	22	75 (30.0%)
Truncated	06	19	05	14	22 (20.4%)	19	14	55 (22.0%)
Assimilated	03	12	02	04	10 (9.3%)	10	06	26 (10.4%)

**Table 2:** Variations in shapes of the lingula between the genders (*n* = 250 sides).

Shape	Male	Female	*p*-Value *
Nodular	42 (30.9%)	52 (45.6%)	0.016 **
Triangular	45 (33.1%)	30 (26.3%)	0.244
Truncated	31 (22.8%)	24 (21.1%)	0.740
Assimilated	18 (13.2%)	08 (7.02%)	0.108
Total	136	114	

* Chi-squared test: (males vs. females); ** statistically significant (*p* < 0.05).

**Table 3:** Height of the mandibular lingula (*n* = 250 sides).

Variable		Male	Female	Total	Min–Max	*p*-Value ^a^	*p*-Value ^b^
Height of the lingula (in mm)	Right	8.05 ± 0.47	7.57 ± 0.34	7.83 ± 0.47	6.89–9.12	0.000 * (z = −5.838)	0.000 * (z = −7.517)
	Left	7.77 ± 0.44	7.48 ± 0.29	7.63 ± 0.41	6.78–8.50	0.000 * (z = −2.955)	

^a^ Mann–Whitney U test (grouping variable: gender); ^b^ Wilcoxon signed-rank test (right vs. left sides); * statistically significant (*p* < 0.05).

**Table 4:** Presence of the lingula and antilingula between the genders by the chi-squared test (*n* = 250 sides).

**Variable**		**Total**	**Gender**		***p*** -Value *
			**Male**	**Female**	
Lingula		250	136	114	0.108
Presence	Count	224	118	106	
	% within the lingula	100%	52.67%	47.3%	
	% within the intragroup	89.6%	86.7%	92.3%	
Absence	Count	26	18	08	
	% within the lingula	100%	69.2%	30.7%	
	% within the intragroup	10.4%	13.2%	7.02%	
Antilingula		250	136	114	0.530
Presence	Count	074	038	036	
	% within the antilingula	100%	51.3%	48.6%	
	% within the intragroup	29.6%	27.9%	31.6%	
Absence	Count	176	98	78	
	% within the antilingula	100%	55.7%	44.3%	
	% within the intragroup	70.4%	72.1%	68.4%	

* Chi-squared test; statistical significance at *p* < 0.05.

**Table 5:** Presence of the lingula and antilingula by the variable of gender and mandibular side.

Variable		Total (*n* = 250)		Male (*n* = 136)		Female (*n* = 114)		*p*-Value *
		Bilateral	Unilateral	Bilateral	Unilateral	Bilateral	Unilateral	
Lingula		108	142	058	078	050	064	0.919
Presence	Count	098	126	052	066	046	060	
	% within the lingula	39.2%	50.4%	20.8%	26.4%	18.4%	24.0%	
	% within the intragroup	90.7%	88.7%	89.6%	84.6%	92.0%	93.7%	
Absence	Count	010	016	06	012	04	04	0.420
	% within the lingula	4.0%	6.4%	2.4%	4.8%	1.6%	1.6%	
	% within the intragroup	9.3%	11.26%	10.3%	15.4%	8.0%	6.25%	
Antilingula		174	076	92	44	82	32	0.247
Presence	Count	036	038	016	022	020	016	
	% within the antilingula	14.4%	15.2%	6.4%	8.8%	8.0%	6.4%	
	% within the intragroup	20.7%	50.0%	17.4%	50.0%	24.4%	50.0%	
Absence	Count	138	038	076	022	062	016	0.756
	% within the antilingula	55.2%	15.2%	30.4%	8.8%	24.8%	6.4%	
	% within the intragroup	19.3%	50.0%	82.6%	50.0%	75.6%	50.0%	

* Chi-squared test.

**Table 6:** Frequency percentage of the prevalence of lingula shapes in different populations.

Mandibular Lingula	Study Design	Population	Triangular (%)	Truncated (%)	Nodular (%)	Assimilated (%)
Tuli et al. (2000) [6]	Dry Mandible	Indian	68.5	15.8	10.9	4.8
Jansisyanont et al. (2009) [5]	Dry Mandible	Thai	29.9	46.2	19.6	4.3
Sekerci et al. (2014) [8]	CBCT	Turkish	14.1	32	51.2	2.7
Senel et al. (2015) [9]	CBCT	Turkish	22	19	32.5	26
Alves et al. (2015) [10]	Dry Mandible	Brazilian	23.3	49	26.5	1.2
Rikhotso et al. (2017) [11]	Dry Mandible	South African	30.8	38.8	21.4	8.9
Jung et al. (2018) [12]	CBCT	South Korean	14.3	29.3	54	2.4
Asdullah et al. (2018) [13]	Dry Mandible	Indian	61.6	46.6	31.6	11.6
Ahn et al. (2020) [14]	CBCT	South Korean	31	25.9	32.8	10.3
Ozalp et al. (2020) [15]	Dry Mandible	Turkish	42	28	30	0
Stipo et al. (2022) [16]	Dry Mandible	Italian	10.8	38.6	26.3	4
Present study (2022)	CBCT	Saudi	30	22	37.6	10

## Data Availability

The data presented in this study are available on request from the corresponding author.
